# The effect of the hexanic extracts of fig *(Ficus carica) *and olive *(Olea europaea)* fruit and nanoparticles of selenium on the immunogenicity of the inactivated avian influenza virus subtype H9N2

**Published:** 2015-09-15

**Authors:** Amir Hossein Asl Najjari, Zolfaghar Rajabi, Mehdi Vasfi Marandi, Gholamreza Dehghan

**Affiliations:** 1 *Department of Clinical Sciences, Faculty of Veterinary Medicine, University of Tabriz, Tabriz, Iran;*; 2 *Department of Avian Diseases, Faculty of Veterinary Medicine, University of Tehran, Tehran, Iran;*; 3 *Department of Biology, Faculty of Sciences, University of Tabriz, Tabriz, Iran*

**Keywords:** Adjuvant, Avian Influenza, Fig, Nano-selenium, Olive

## Abstract

Influenza is a contagious viral disease that is seen in avian, human and other mammals, so its control is important. Vaccination against influenza virus subtype H9N2 is one of the ways in controlling program, for this reason several vaccines has been produced. Recently, application of inactivated oil-emulsion vaccines in poultry for controlling low pathogenic avian influenza is increasing. At present, oils that are used as adjuvant in commercial vaccines are mineral oils, which not only lack immunizing effect, but also produce some detriments. The aim of this study is the evaluation the immunogenicity of vegetable oils, which are more metabolizable and safer than mineral oils. In this study the efficacy of hexanic extracts of fig* (Ficus carica) *and olive* (Olea europaea)* fruit and also nano-selenium on the immunogenicity of the inactivated avian influenza virus subtype H9N2 was evaluated in broiler chickens. The results indicated that the prepared emulsions could elicit a little degree of immunity, but they could not inhibit the anamnestic response and infection. With regard to the results, it seems that the intact mixture of fig and olive fruit hexanic extracts could not be administered as an immunoadjuvant in the vaccine, and about nano-selenium. In spite of positive effect on the immunogenicity of avian influenza virus subtype H9N2, it still needs more work.

## Introduction

Avian influenza (AI) as a contagious viral disease in wide variety of birds and human, is caused by viruses in the family *Orthomyxoviridae*. Outbreaks due to H9N2 subtype occurred in domestic poultry during 1994-1999 in most of the countries as well as Iran.^1^^,^^2^^,3^Relationship between internal genes of H5N1 and H9N2 subtype,^4^ and isolation of H9N2 from human being makes important controlling of this subtype.^5^^,^^6^ Vaccination against influenza virus subtype H9N2 is one of prevention and controlling program, for this reason several vaccines have been produced, and recently, administration of inactivated oil-emulsion vaccines in poultry for controlling avian influenza (especially low pathogenic avian influenza) is increasing.

In formulation of a vaccine, adjuvants are essential.^7 ^Light mineral oils used as adjuvant in preparation of many efficacious commercial oil-emulsion poultry vaccines, but they have various disadvantages such as persistence in tissues, excessive tissue reactions, carcinogenic feature, risk of accidental self-injection by vaccinators and long withdrawal-time.^8^^-^^12^ Some studies indicated that animal- and vegetable-oil vaccines have potential in replacing mineral-oil vaccines.^11^ The present study describes the efficacy of hexanic extracts of fig (*Ficus carica*) and olive (*Olea europaea*) fruit as substitutes for the mineral oil currently used in water-in-oil formulations. Also, in this study the immunogenicity of nanoparticles of selenium as an immune potential compound in combination with fruit extracts was investigated.

## Materials and Methods

**Experimental procedures**. In this study 50 one-day-old mixed-sex (Cobb^®^ 500) broiler chickens were used in the experiment. All the chickens were reared in battery cages under the same environment and management according to Cobb broiler catalogue. ^13^

Chickens were randomly divided into five groups (10 chickens per group). Each group contained two replicates (five chickens per replicate). Group 1 and 2 received the emulsion 1 and 2 (0.5 mL per chick) respectively; group 3 received mixed inactivated AI virus, in equivalent level as described in first and second emulsions, with equal part of sterile PBS; group 4 received Commercial mineral-oil AI vaccine (Razi Vaccine and Serum Research Institute, Karaj, Iran) as a known standard control vaccine (0.2 mL per chick as manufacturer’s instruction); and group 5 was a control group without any injection. The injection was done subcutaneously at 13 days old. Serum samples were collected weekly for hemagglutination-inhibition (HI) tests between 1 to 3 weeks post-vaccination before they were challenged with the strain of avian influenza virus (AIV).

All groups challenged at three weeks post-vaccination.

The low pathogenic strain of AIV (A/Chicken/Iran/ZMT- 101(101)/98(H9N2)) at 10^-1^ dilution was applied in 0.1 mL volume by intranasal and eye drop (the amount of applied virus was equal in all solutions and the emulsions). Survivors were bled 14 days after challenge for HI serology to determine the secondary serologic responses (anamnestic response), and also clinical signs were recorded.

**Virus propagation**. Avian influenza virus, A/Chicken/ Iran/ZMT-101(101)/98(H9N2), was diluted 1/10 in sterile PBS (Merck, Darmstadt, Germany), then 0.1 mL of the solution was injected into the allantoic sacs of 10 chicken eggs (10-day-old embryonated). Eggs were incubated at 37 ˚C for six days. During incubation, each egg was candled daily to determine embryo viability before chilling at 4 ˚C. Allantoic fluid that harvested from each egg, tested for hemagglutination assay (HA).

**Preparation of whole virion antigen.** Inactivation of the purified virus was performed by mixing the virus with 0.1% formalin at room temperature for 4 hr. Inactivation of the virus was confirmed by inoculation of the formalin-treated virus into the allantoic sacs of 10-day-old embryonated chicken eggs and virus replication was measured by the HA. The inactivated virus preserved with 0.01% thimerosal, and frozen at –70 ˚C.

**Extract preparation**. The fruits separately were dried at room temperature for one week, and then crushed. Obtained powder was macerated into hexane (Merck, Darmstadt, Germany) for one day and this process repeated three times. After filtration, the filtrate was evaporated by using vacuum evaporator.

**Nano-selenium prepration. **The nano-selenium was prepared in Institue of Biophysic and Biochemistry, University of Tehran, Tehran, Iran. 

**Preparation of emulsions. **For this purpose, we prepared two emulsions: 

1. Mixture of hexanic extract of olive and fig fruits and inactivated H9N2 subtype: The emulsions were formulated with one part inactivated AI (HA titer was 7) virus and one part mixed hexanic extract of olive and fig fruits (½ fig extract and ½ olive extract). The emulsions were made one day before use. Mixture of the extracts and suspension of inactivated H9N2 subtype homogenized at 9000 rpm for 5 min at 4 to 8 ˚C by the Homogenizer (IKA Ultra-Turax^®^ T 18 basic; IKA, Staufen, Germany). The emulsion also had 10% Span80 and 1.0% Tween80 as an oil-soluble and water-soluble surfactant in its compound.

2. Mixture of hexanic extract of olive and fig fruits, inactivated H9N2 subtype and nano-selenium: In addition to hexanic extract of olive and fig fruits and H9N2 subtype, nano-selenium (0.1 mg per dose), which was approximately 10-fold of the original treatment dose,^14^ was added to the emulsion, and homogenized as described before. The amount of applied extracts and H9N2 subtype was equal in the two emulsions.

**Vaccine efficacy tests.** Efficacy of the vaccines was determined by serology and resistance to challenge by measuring anamnestic response.^15^ An anamnestic response with a four fold-rise in antibody titre is indicative of recent infection in vaccinated chickens.

**Serology.** Hemagglutination-inhibition antibody levels in serum were determined by using four HA units of AIV antigen. HI tests were conducted the day that serum samples were taken.^16^

**Statistical analysis. **One-way ANOVA and Duncan multiple range tests were applied for determining differences in mean HI titers of treatment and control groups from three weeks post-vaccination. The SPSS statistics (Version 22; SPSS Inc., Chicago, USA) was used for statistical analysis.

## Results

**Fig. 1:**
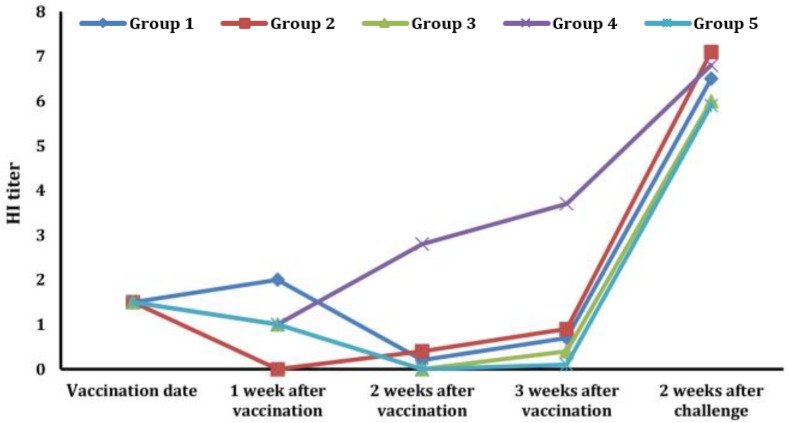
Mean hemagglutination inhibition (HI) titer of the chickens. Group 1: Chickens that received the emulsion containing hexanic extract of olive and fig fruit and inactivated H9N2 subtype. Group 2: Chickens that received the emulsion containing hexanic extract of olive and fig fruit, inactivated H9N2 subtype and nano-selenium. Group 3: Chickens that received the mixture that contained inactivated H9N2 subtype and PBS. Group 4: Chickens that received avian influenza commercial vaccine. Group 5: Chickens that did not received anything, but challenged as the other groups. The mean HI titers of different groups indicate that the prepared emulsions could elicit a little degree of immunity, but in contrast to group 4, they could not inhibit the anamnestic response and infection

**Serology. **Mean HI titers in all groups on 13-day-old chickens were 1.5. After 1 week of vaccination, the mean HI titers of groups 1, 2, 3, 4, and 5 were 2.0, 0, 1.0, 1.0, 1.0, respectively; statistically there were significant differences between group 2 and other groups and also between group 1 and other groups (*p* < 0.05). Two weeks post-vaccination, the mean HI titers of groups 1, 2, 3, 4, and 5 were 0.2, 0.4, 0, 2.8, and 0, respectively. Statistically, there were significant differences between group 4 and other groups, and also between group 2 and groups 3, 4, and 5 (*p* < 0.05). After three weeks of vaccination the mean HI titers of groups were 0.7, 0.9, 0.4, 3.7 and 0.1 that statistically there were significant differences between group 5 and groups 1, 2, and 4, and also between group 4 and other groups (*p* < 0.05). The mean HI titer of group 4 at weeks 1 and 2 post-vaccinationshowed the highest levels, 2.8 and 3.7, respectively. The HI titer of group 5 was minimum. After challenging with AIV, the mean HI titers of groups 1-5 reached 6.5, 7.1, 6.0, 6.8, and 5.9, respectively, in 48 days old chicks that statistically there were not significant differences between them (Fig. 1).

**Clinical signs. **After several days of challenge, some chickens in all groups, except group 4, exhibited, depression, ruffled feathers, conjunctivitis, sneezing, rales, green diarrhea, and also loss of appetite, but no chickens were died after challenge. Clinical signs in group 5 were more severe than other affected groups.

## Discussion

It has been shown that herbal adjuvants are able to increase efficacy of vaccines^17^ and some results indicate that both Ginseng and Salviae play a role as mucosal adjuvants against influenza virus as well as immuno-modulators during influenza virus infection.^18^ Stone showed that an inactivated avian influenza vaccine formulated from peanut oil induced protection against morbidity and death when vaccinated chickens were challenged with a virulent isolate of avian influenza virus. Hemagglutination-inhibition titers induced by mineral-oil vaccines were not significantly different from those induced by the most efficacious formulations prepared from animal and vegetable oils. Tissue reaction from injection of animal- and vegetable-oil vaccines was less than that induced by mineral-oil vaccines.^11^ Lianju *et al*. showed that the nontoxic extracts of fig could increase the immunity of the bodies and postponed the life span after the mice were irradiated by Cobalt-60.^19^

Important role of the selenium on immune system of the body is obvious and the most available form of this trace element is selenite. Several studies have shown that bioavailability of nano-selenium is such as selenite, mean-while its toxicity is less than selenit.^20^^-^^22^ Nanoparticles represent promising delivery systems for a number of applications especially after parenteral and peroral administration.^23 ^The addition of nano selenium in the feed of laying chicks could promote their growth and strengthen the function of their immunity organs.^24^

The anamnestic serologic response depends on only infection of the chicken when the chicken is already primed by that antigen. Therefore, the anamnestic response would be respected to be a more sensitive and reliable indicator of virus infection than virus isolation.^15^ Lack of an anamnestic response is an indirect evidence of resistance to infection and therefore prevention of virus shedding. The anamnestic response following challenge appears to be the ultimate parameter to measure the effectiveness of a vaccine.^15^ The absence of a detectable anamnestic response after challenge supports the contention that replication was limmited in these birds after challenge.

The weekly (serial serum sampling) mean HI titer of group 5 indicates that the control and treatment groups were not contaminated with H9N2 subtype of avian influenza virus before challenge.

Inactivated vaccines usually induce peak antibody leves 2 to 4 weeks after vaccination,^25^ especially in primed chickens; in this study the mean HI titers of the chickens before vaccination and after one week of vaccination refer maternal antibodies, because the breeders of the chickens had been vaccinated against avian influenza subtype H9N2. After two weeks of vaccination mean HI titers of group 1 and 2 were higher than group 3, which about group 2 mean HI titer was significantly higher than the later group. This means not only good effect of hexanic extract of olive and fig fruits on increasing of immunogenicity of inactivated H9N2 subtype, but also confirms the positive effect of nano-selenium on the immunogenicity. The comparison of mean HI titers of different groups after three weeks of vaccination statistically indicates positive effects of the extracts and nano-selenium on the immunogencity of inactivated H9N2 subtype as mentioned above. After challenge there were not significantly differences between groups, but groups 1 and 2, especially group 2, which in addition the extracts contains nano-selenium, in spite of group 4, which had been received commerial AI vaccine, were not able to prevent chickens from disease and infection, because some of the chickens clinically showed signs of disease and serologically mean HI titers of them had been increasesd approximately 9.3- and 7.9- fold at two weeks after challenge, in contrast to group 4 that increased lower than 2-fold. Therefore, chickens in treatment groups, group 1 and 2, had anamnestic responses as chickens in control group, group 3. Anamnestic response implyes occuronce of infection in challenged birds of all groups other than group 4. It seems that the presence of fatty acids in fig extract^26^ is the reason of positive effect of the extract on the immune responses. 

It has been known that linoleic and linolenic acid can convert to hormone-like substances called eicosanoids which affect physiological reactions ranging from blood clotting to immune response,^27^ but perhaps the presence of phytostrols and oleic acid in fig extract,^26^ and also hydroxytyrosol (HT)^28^ and oleic acid in olive extract decrease the positive effect of linoleic and linolenic acid on the immune responses. Eugster et al. showed antiviral effect of phytosterols on HIV-1, human cytomegalovirus (HCMV) and herpes simplex virus (HSV).^29^ It has been known that HT can disrupt H9N2 virus,^30^ and also about oleic acid which is the main monounsaturated fatty acid of the olive and fig extract has antiviral activity on Semliki Forest virus.^31^ Therefore, it seems that the inactivation of the antiviral components of olive and fig extracts may increase the immunogenicity of the extracts against avian influenza virus subtype H9N2.

In conclusion, although, each of the olive and fig hexanic extract had immunogenicity effect, the intact extracts could not be used as immunoadjuvants in formulation of vaccine prepration, and about nano-selenium. In spite of positive effect on the immunogenicity of avian influenza virus subtype H9N2, it still needs more work.
